# New Considerations for Collecting Biomechanical Data Using Wearable Sensors: The Effect of Different Running Environments

**DOI:** 10.3389/fbioe.2020.00086

**Published:** 2020-02-14

**Authors:** Lauren C. Benson, Christian A. Clermont, Reed Ferber

**Affiliations:** ^1^Faculty of Kinesiology, University of Calgary, Calgary, AB, Canada; ^2^Running Injury Clinic, Calgary, AB, Canada; ^3^Faculty of Nursing, Cumming School of Medicine, University of Calgary, Calgary, AB, Canada

**Keywords:** running, machine learning, classification, treadmill, outdoor

## Abstract

Traditionally, running biomechanics analyses have been conducted using 3D motion capture during treadmill or indoor overground running. However, most runners complete their runs outdoors. Since changes in running terrain have been shown to influence running gait mechanics, the purpose of this study was to use a machine learning approach to objectively determine relevant accelerometer-based features to discriminate between running patterns in different environments and determine the generalizability of observed differences in running patterns. Center of mass accelerations were recorded for recreational runners in treadmill-only (*n* = 28) and sidewalk-only (*n* = 25) environments, and an independent group (*n* = 16) ran in both treadmill and sidewalk environments. A feature selection algorithm was used to develop a training dataset from treadmill-only and sidewalk-only running. A binary support vector machine model was trained to classify treadmill and sidewalk running. Classification accuracy was determined using 10-fold cross-validation of the training dataset and an independent testing dataset from the runners that ran in both environments. Nine features related to the consistency and variability of center of mass accelerations were selected. Specifically, there was greater ratio of vertical acceleration during treadmill running and a greater ratio of anterior-posterior acceleration during sidewalk running in both the training and testing dataset. Step and stride regularity were significantly greater in the treadmill condition for the vertical axis in both the training and testing dataset, and in the medial-lateral axis for the testing dataset. During sidewalk running, there was significantly greater variability in the magnitude of the vertical and anterior-posterior accelerations for both datasets. The classification accuracy based on 10-fold cross-validation of the training dataset (*M* = 93.17%, SD = 2.43%) was greater than the classification accuracy of the independent testing dataset (*M* = 83.81%, SD = 3.39%). This approach could be utilized in future analyses to identify relevant differences in running patterns using wearable technology.

## Introduction

Traditional running biomechanical analysis is confined to treadmill or over-ground indoor running (Simon, 2004). In contrast, most runners complete their runs outdoors (Taunton et al., 2003) and research has shown that machine learning algorithms trained on gait-related features from an accelerometer can distinguish whether a runner is running on concrete, synthetic, woodchip surfaces (Schütte et al., 2016). However, to our knowledge, no study has examined differences in running biomechanics between indoor running, where the speed, surface inclination and available space are often dictated by a treadmill or a small flat runway, and outdoor running, where these features are less controlled. Insights gleaned from biomechanical analyses conducted in less controlled settings may be more applicable to runners who train and compete outdoors.

Limited research has been conducted to compare treadmill to overground running, but has shown that the running biomechanical patterns during treadmill running gait dynamics do not necessarily reflect overground running patterns (Lindsay et al., 2014; Schütte et al., 2016). Moreover, methodological limitations make it difficult to generalize these results. For example, Lindsay et al. (2014) compared treadmill running to overground running on an indoor track and Schütte et al. (2016) performed outdoor investigations on a short, flat and straight course, limiting the ability to generalize the findings to runners outside of the study sample and real-world conditions. Dixon et al. (2019) collected only 8 s of data, from between 2 and 4 running trials, whilst runners ran on a straight 90 m segment of either concrete road, synthetic track, or woodchip trail. Indoor tracks and short, straight and flat runways do not necessarily reflect real-world running conditions, particularly for long-distance runners. Thus, research is needed in order to collect running biomechanical data in a runner’s natural environment. Considering that the vast majority of running biomechanical data collected to date have been in controlled laboratory settings, it will be beneficial to understand which biomechanical variables are similar, or dissimilar, to those exhibited during running in real-world environments.

Inertial measurement units (IMUs) are portable devices that can be used to quantify running biomechanical patterns in a runner’s natural environment (Norris et al., 2014; Reenalda et al., 2016), yet, these investigations are still rare (Benson et al., 2018a). Running biomechanical analysis using IMUs is commonly conducted by recording 3D center of mass accelerations and extracting features related to the magnitude, consistency and variability of the signal (Henriksen et al., 2004; Moe-Nilssen and Helbostad, 2004; Kobsar et al., 2014; Benson et al., 2018b; Clermont et al., 2018). There remains an absence of an association between joint-level mechanics commonly investigated using laboratory-based motion capture systems and features generated from center of mass accelerations. Thus, a challenge in identifying new methods for collecting biomechanical data using wearable sensors is to identify which accelerometer-based features are relevant for observing running patterns in real world settings.

The purpose of this study was to determine whether running environments could be successfully classified from movement patterns quantified by the use of a single accelerometer, with generalizability to an independent dataset. A secondary objective was to determine which features drive successful classification between treadmill-only and sidewalk-only running. It was expected that key features would quantify the consistency and variability of running patterns, and that the model would be generalizable to an independent set of runners.

## Materials and Methods

### Participants and Equipment

A total of 69 self-identified recreational runners provided informed consent to participate in this study approved by the Ethics Board at the University of Calgary (REB16-1183). Both male and female runners with no running-related injury in the previous 6 months were included. All participants were outfitted with an IMU (Shimmer3 GSR+^®^ ±8 g, Shimmer Inc., Dublin, IE, United States) on the lower back near the center of mass, such that the positive *x*-axis pointed to the right, the positive *y*-axis pointed vertically, and the positive *z*-axis pointed posteriorly. Three-dimensional accelerations were recorded at 201.03 Hz and stored on an SD card. Additionally, a GPS-capable watch (Garmin vivoactive HR, Garmin Inc., Olathe, KS, United States) with a sampling rate of 1 Hz was worn on the preferred wrist. Participants wore their preferred clothes and shoes.

### Data Collection

**FIGURE 1:**
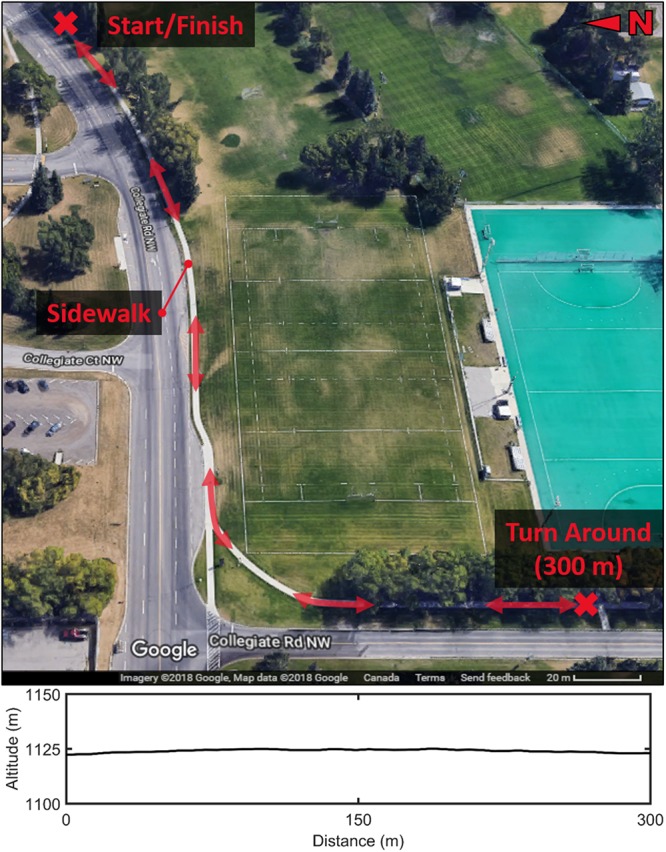
Map of outdoor running path (300 m from start to turn around) and associated altitude along path.

Each participant was included in just one of three protocols, based on weather (i.e., outdoor running only occurred on days with no snow or rain) and availability to attend multiple sessions (Table 1). In Protocol 1, 28 participants ran on a level treadmill (Bertec, Columbus, OH, United States) only. The speed was initially set to a speed equal to what the participant self-reported as their typical training pace, and was subsequently adjusted in 0.1 m/s increments until it matched the participant’s preferred speed, described as “a pace which you would be comfortable to run for about 45 min and represents a usual, common, or typical pace (Lindsay et al., 2014).” Participants first completed a 5–10 min warmup at this speed. Next, data were recorded as the participants ran at their preferred speed (recorded as the treadmill setting) for 5 min. In Protocol 2, 25 different participants ran outdoors on a concrete sidewalk only. First, participants completed a 5–10 min warmup at their own pace. Then, data were recorded as the participants ran at their preferred running speed (recorded with GPS watch) on a continuous stretch of sidewalk that featured a straightaway, curve and slight incline typical of real-world outdoor running conditions. The sidewalk was 300 m, and the participants paused for 10 s at the turnaround to complete a total of 600 m (Figure 1). It was expected that all runners would complete the 600 m course within 5 min (8:20/km pace). In Protocol 3, a different set of 16 participants completed both the treadmill and sidewalk runs on separate days, with the order of days randomized, via a coin flip, for each participant.

**TABLE 1:** Mean (SD) descriptive variables for each protocol.

	**Protocol 1**	**Protocol 2**	**Protocol 3**

**Environments**	**TM (5 min) only**	**S (600 m) only**	**TM (5 min) and S (600 m)**
Sex	18M, 10F	12M, 13F	8M, 8F
Height, m	1.74 (0.09)	1.73 (0.10)	1.70 (0.09)
Mass, kg	70.5 (10.3)	70.2 (13.0)	67.1 (8.1)
Age, yr	32.2 (13.4)	36.9 (10.1)	31.3 (10.2)
TM speed, m/s	2.78 (0.26)	–	2.75 (0.39)*
S speed, m/s	–	3.24 (0.42)	3.10 (0.60)*

TM, treadmill; S, sidewalk. * Within Protocol 3, TM speed was significantly lower than S Speed, *p* = 0.001. There were no significant differences between protocols for any variables.

### Data Processing

For each run, the accelerometer data were filtered using a 4th-order low-pass Butterworth filter with a cutoff frequency at 10 Hz (Wundersitz et al., 2015), and the first and last 5% of the signal was removed to eliminate effects of starting and stopping. The trimming was applied to each 300 m section of the sidewalk runs, as a complete turnaround is likely not generalizable to real-world running conditions. The acceleration signal was then aligned with gravity (Moe-Nilssen, 1998) and the direction of motion within the horizontal plane (Avvenuti et al., 2013). The signal was segmented into steps (Lee et al., 2010), each step was normalized to 50 data points, and a previously defined set of 24 features (Moe-Nilssen and Helbostad, 2004; Kobsar et al., 2014; Barden et al., 2016) was extracted from the signal (Table 2). These features included the peaks, magnitude (RMS), and ratio of the acceleration in three dimensions, averaged across all steps. Several features related to consistency and variability of the running pattern across all steps and strides. Regularity is the consistency of the stride-to-stride or step-to-step pattern, while symmetry is the difference between step and stride regularity (Barden et al., 2016), and higher values indicate a more consistent gait pattern. Mean running speed was included as a 25th feature for each participant.

**TABLE 2:** All features extracted from the accelerometer signal for each participant and running condition.

**Feature**	**Independent of axes**	**AP**	**ML**	**VT**
Speed*	✓			
Step time CV	✓			
Stride time CV	✓			
RMS tesultant	✓			
Regularity step		✓	✓	✓
Regularity stride		✓	✓	✓
Symmetry (regularity step/regularity stride)		✓	✓	✓
Peak		✓	✓	✓
RMS		✓	✓	✓
RMS CV		✓	✓	✓
Ratio (RMS/RMS resultant)		✓	✓	✓

AP, anterior-posterior axis; ML, medial-lateral axis; VT, vertical axis; CV, coefficient of variation; RMS, root mean squared. *Speed was determined from the GPS watch or treadmill setting, not the accelerometer signal.

### Feature Selection

**FIGURE 2:**
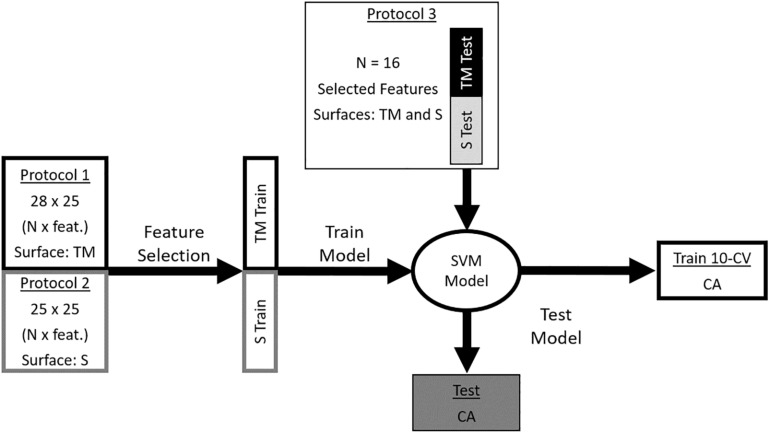
The data from Protocol 1 and Protocol 2 were used to create a model to distinguish treadmill running from sidewalk running. Prior to building the model, the number of features in the training dataset was reduced following a feature selection task. The two environments from Protocol 3 were used as an independent testing dataset for the model. The features in the testing dataset matched the selected features in the training dataset. TM, treadmill; S, sidewalk; SVM, support vector machine; CA, classification accuracy; 10-CV, 10-fold cross-validation of the training dataset.

To improve generalizability of classification and to reduce model complexity, a subject-specific forward-sequential feature selection algorithm with a linear discriminant analysis wrapper and 10-fold cross-validation (Chizi and Maimon, 2010; Caby et al., 2011) was applied to the data from Protocols 1 and 2 to identify relevant features, ranked based on their order of selection, for the classification of running environments (Figure 2). Only the features selected in at least 10% of 100 iterations were retained, and the selected features in Protocols 1 and 2 became the training dataset. All data processing and feature selection was done using custom MATLAB software (v9.1.0.441655, Mathworks, Inc., Natick, MA, United States).

### Classification

The training dataset was used to train a binary support vector machine classifier (Shmilovici, 2010) for treadmill and sidewalk, with all hyper-parameters optimized with the MATLAB function *fitcsvm*. The model was tested two ways: (1) 10-fold cross-validation of the training dataset from Protocol 1 and 2, with each participant’s data in only one fold at a time, and (2) the selected features from both runs in Protocol 3 were used as an independent testing dataset. The classification process was repeated for 100 iterations, and an average classification accuracy across all iterations was determined.

### Statistical Analysis

Height, mass, age, and treadmill or sidewalk speed were checked for normality and compared across protocols in separate ANOVAs. A paired *t*-test was used to detect differences in speed between treadmill and sidewalk among participants within Protocol 3. Differences between treadmill and sidewalk for each of the selected features were determined with independent *t*-tests for the training dataset and paired *t*-tests for the testing dataset. For each statistical test, significance was determined at *p* < 0.05, with a Bonferroni adjustment based on number of comparisons. All statistical analyses were done using SPSS (v24.0.0.1, SPSS, Inc., Chicago, IL, United States).

## Results

There was no significant effect of protocol for height, mass, age, or treadmill or sidewalk speed (*p* > 0.05). Within Protocol 3, speed was significantly different (*p* = 0.001) between treadmill running (*M* = 2.75 m/s, SD = 0.39 m/s) and sidewalk running (*M* = 3.10 m/s, SD = 0.60 m/s).

**FIGURE 3:**
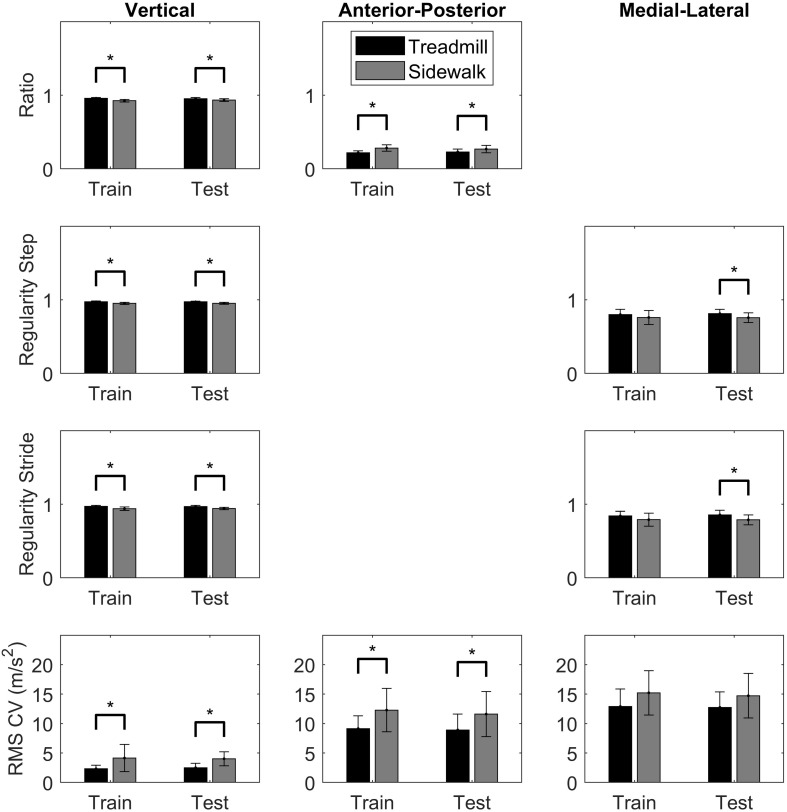
Comparisons between treadmill (black) and sidewalk (gray) conditions for each of the nine selected features used in the model. Independent *t*-tests were used for the training dataset comparisons and paired *t*-tests were used for the testing dataset comparisons. Since a total of 18 comparisons were made, significance (*) was determined at *p* < 0.003.

Nine features were selected to discriminate treadmill and sidewalk running (Table 3 and Figure 3). There was a greater ratio of vertical acceleration during treadmill running and a greater ratio of anterior-posterior acceleration during sidewalk running in both the training and testing dataset. Step and stride regularity were significantly greater in the treadmill condition for the vertical axis in both the training and testing dataset, and in the medial-lateral axis for the testing dataset. During sidewalk running, there was significantly greater variability in the magnitude of the vertical and anterior-posterior accelerations for both datasets.

**TABLE 3:** Selected features used in the classification model.

**Mean rank**	**Selected features**
1.00	Ratio VT
1.05	Ratio AP
2.06	Regularity step ML
2.06	RMS CV ML
2.30	Regularity stride VT
2.39	RMS CV AP
2.67	RMS CV VT
2.86	Regularity stride ML
3.00	Regularity step VT

*AP, anterior-posterior; ML, medial-lateral; VT, vertical; CV, coefficient of variation; RMS, root mean squared. Features were ranked according to the order in which they were selected during the 10-fold cross-validation of the feature selection algorithm, and the mean rank over 100 iterations of feature selection is reported for features selected at least 10% of the time.*

The initial classification accuracy based on 10-fold cross-validation of the training dataset (*M* = 93.17%, SD = 2.43%) was greater than the classification accuracy of the independent testing dataset (*M* = 83.81%, SD = 3.39%). Over 100 iterations, ten participants had both conditions correctly classified at least 82 times, and the remaining six had poor classification of one condition but perfect classification of the other condition (Table 4).

**TABLE 4:** Number of correctly predicted environments for each participant in the testing dataset over 100 iterations (max = 100).

**Test participant**	**Number correct treadmill predictions**	**Number correct sidewalk predictions**
1	100	1
2	100	21
3	100	25
4	100	95
5	100	96
6	100	100
7	100	100
8	100	100
9	99	100
10	92	98
11	87	99
12	84	97
13	82	100
14	53	100
15	40	100
16	13	100

## Discussion

The purpose of this study was to classify running environments based on features extracted from a single accelerometer and identify features that would represent the difference between treadmill and sidewalk running. Sidewalk running was characterized by lower regularity and greater variability than treadmill running and using these features, classification accuracy over 80% was achieved for both the training dataset and an independent dataset. These results are supported by Lindsay et al. (2014) who also reported that the treadmill running requires greater constraints and increased voluntary control during running gait. Thus, researchers must use caution when generalizing laboratory-based treadmill running results to real-world conditions for purposes such as rehabilitation of injuries, improved performance, and/or injury prevention (Benson et al., 2018a).

The observed changes in running patterns in different running environments are likely due to the consistency of the surfaces and/or speed in each environment. For example, a treadmill offers a smooth and consistent running surface and a constant speed for every step, whereas outdoor running presents more variable conditions with opportunities for changes in speed, surface, inclination, turns in the running path, other pedestrians/runners, and/or changes in weather or temperature (Ahamed et al., 2017, 2018; Benson et al., 2019). This lack of consistency likely contributed to the decrease in regularity in the vertical and medial-lateral dimensions, and changes in the ratios of the magnitude of the acceleration. The decrease in regularity and observed shift to a greater ratio of horizontal accelerations than vertical accelerations when on sidewalk is consistent with previous research that has shown differences between stable and unstable surfaces based center of mass accelerations (Menz et al., 2003; Schütte et al., 2016) and stride time analyses (Lindsay et al., 2014). Sidewalk running was also characterized by greater variability in the magnitude of accelerations in all three dimensions. From a dynamical systems approach, a lack of coordinative variability in movement patterns may be associated with an unhealthy or pathological state (Hamill et al., 2012). However, the current study did not calculate coordinative variability in a manner similar to the methods proposed by Hamill et al. (2012), so future prospective studies should consider a link between the increased center of mass variability observed during sidewalk running and running-related injuries.

Due to the influence of speed on the magnitude of center of mass accelerations (Kobsar et al., 2014; Benson et al., 2018b), and the tendency to preferentially select a slightly slower speed during treadmill compared to overground running (Kong et al., 2012), speed was included as a potential feature in the classification model. However, speed was not one of the selected features used in the model. Therefore, differences in features related to the variability and consistency of the accelerometer signal had a greater role in discriminating between treadmill and sidewalk running.

The ability to generalize these results beyond the current study may be influenced by overfitting the classification model to the study participants (Ferber et al., 2016). Despite the use of 10-fold cross-validation of the training dataset to attempt to improve generalizability of classification, the model slightly overfit to the training dataset as there was lower classification accuracy for the independent testing dataset compared to the 10-fold cross-validation of the training dataset. Regarding real-world usability, previous studies that have classified IMU-generated running and walking patterns have consistently reported classification accuracy greater than 80% (Kobsar et al., 2014, 2015; Phinyomark et al., 2014; Ahamed et al., 2018, 2019; Benson et al., 2018b; Clermont et al., 2018). Thus, the reported 93.17% accuracy for the training dataset and 83.81% accuracy for the independent testing dataset in the current study suggests that this classification mechanism has practical use.

The nearly 10% difference in classification accuracy between the training and testing datasets can be attributed to differences in running patterns between individuals in each dataset. In the cases where an individual in the testing dataset had a low classification rate for one environment, there was a perfect classification rate for the other environment. This result does not suggest that these misclassified participants have the same running pattern in both environments, but rather their running pattern on one environment is similar to the running patterns of other runners on the opposite. For example, the poor treadmill classification for test participant 16 (Table 4) was most likely driven by anterior-posterior variability in the treadmill condition that was greater than the sidewalk anterior-posterior variability for all participants in the training dataset. Yet, test participant 16 had perfect classification accuracy in the sidewalk condition as their anterior-posterior variability in the sidewalk condition was even greater than their treadmill value. Therefore, the misclassifications observed in this study highlight the potential strength of subject-specific models of running biomechanics to monitor changes in an individual’s running biomechanics (Ahamed et al., 2018, 2019; Benson et al., 2019) and should be further investigated in future studies.

In addition to the previous limitations discussed, other limitations are acknowledged. First is the possibility that other unmeasured variables may also differ between running environments. The measured variables were previously used to quantify running patterns and were thus considered suitable for this study. However, *a priori* variable selection suggests a risk of investigator bias and may lead to the dismissal of potentially meaningful information that could be represented by other variables, such as metrics related to the accelerometer signal frequency content. Second, in addition to other accelerometer-based features, physiological metrics such as heart rate may differ between running environments. A further limitation is that although many of the features used in this study were on a scale of 0–1 (e.g., ratio of acceleration in a given axis, symmetry, regularity), other features were not on the same scale which may have influenced the contribution of each variable in the classification model. Nevertheless, six of the nine selected features, and four of the top-five features, were on the 0–1 scale, suggesting that features with values greater than 1 did not have an undue influence on the classification model.

In conclusion, we used a machine learning approach to successfully select features related to the consistency and variability of center of mass accelerations between treadmill and sidewalk running. Overall, step and stride regularity were significantly greater during treadmill running while sidewalk running resulted in significantly greater variability in the magnitude of the vertical and anterior-posterior accelerations. Based on a 10-fold cross-validation of the training dataset we achieved a 93.17% classification accuracy, which was greater than the 83.81% classification accuracy of the independent testing dataset. The overall machine learning approach presented here could be utilized in future running biomechanical analyses to identify relevant differences in running patterns using IMUs.

## Data Availability Statement

The datasets generated for this study are available on request to the corresponding author.

## Ethics Statement

The studies involving human participants were reviewed and approved by the Conjoint Human Research Ethics Board (CHREB) at the University of Calgary (REB16-1183). The patients/participants provided their written informed consent to participate in this study.

## Author Contributions

RF, LB, and CC designed the study and were responsible for writing the manuscript. LB and CC were responsible for the data collection and data analysis.

## Conflict of Interest

The authors declare that the research was conducted in the absence of any commercial or financial relationships that could be construed as a potential conflict of interest.
